# Transcriptomic DN3 clock neuron subtypes regulate *Drosophila* sleep

**DOI:** 10.1126/sciadv.adr4580

**Published:** 2025-01-03

**Authors:** Dingbang Ma, Jasmine Quynh Le, Xihuimin Dai, Madelen M. Díaz, Katharine C. Abruzzi, Michael Rosbash

**Affiliations:** ^1^ Interdisciplinary Research Center on Biology and Chemistry, Shanghai Institute of Organic Chemistry, Chinese Academy of Sciences, Shanghai 201210, China.; ^2^ Shanghai Key Laboratory of Aging Studies, Shanghai 201210, China.; ^3^ Howard Hughes Medical Institute, Brandeis University, Waltham, MA 02453, USA.; ^4^ Department of Biology, Brandeis University, Waltham, MA 02453, USA.; ^5^ Department of Psychology, Florida International University, Miami, FL 33199, USA.

## Abstract

Circadian neurons within animal brains orchestrate myriad physiological processes and behaviors, but the contribution of these neurons to the regulation of sleep is not well understood. To address this deficiency, we leveraged single-cell RNA sequencing to generate a comprehensive census of transcriptomic cell types of *Drosophila* clock neurons. We focused principally on the enigmatic DN3s, which constitute most fly brain clock neurons and were previously almost completely uncharacterized. These DN3s are organized into 12 clusters with unusual gene expression features compared to the more well-studied clock neurons. We further show that previously uncharacterized DN3 subtypes promote sleep through a G protein–coupled receptor, *TrissinR*. Our findings indicate an intricate regulation of sleep behavior by clock neurons and highlight their remarkable diversity in gene expression and functional properties.

## Teaser

A comprehensive gene expression census of *Drosophila* clock neurons identifies DN3 subtypes that regulate sleep.

## INTRODUCTION

A comprehensive characterization of transcriptomic neuron types, their projection patterns, and how they give rise to different complex behaviors are major goals of contemporary neuroscience research. The circadian clock and its brain neurons are ideal substrates to help achieve these aims (*1*, *2*). The clock is regulated by a transcription-translation feedback loop, which resides within these brain clock neurons and times different rhythmic behaviors of animals (*3*, *4*).

The principal circadian pacemaker of mammals is localized in the suprachiasmatic nucleus (SCN) in the brain. Each unilateral SCN of the mice brain contains about 10,000 neurons, which have been classically divided into two main regions based on the expression of two neuropeptides: the vasoactive intestinal polypeptide expressing core and arginine vasopressin expressing shell (*5*). Recent single-cell RNA sequencing studies have revealed even more heterogeneity. There are for example at least five different neuronal cell types in the SCN (*6*–*8*). Relevant to sleep regulation is the innervation of the dorsomedial hypothalamic nucleus (DMH) by SCN processes: The DMH inhibits the activity of sleep-promoting ventrolateral preoptic neurons and activates the activity of wake-promoting hypocretin neurons via different neurotransmitters (*9*–*12*).

Similar to the SCN, the circadian clock of *Drosophila* is regulated by functionally and morphologically diverse clock neurons in the brain. Recent *Drosophila* FlyWire brain connectome analysis revised the number of clock neurons from 150 to 240 (*13*). On the basis of the anatomical locations of their cell bodies and their projection patterns, most of the newly annotated neurons are DN3s. The DN3s are one of the dorsal circadian neuron groups that also includes the DN1a, DN1p, and DN2. There are also five lateral neuron groups including the small ventral lateral neurons (s-LNv), the large ventral lateral neurons (l-LNv), the dorsal lateral neurons (LNd) and the lateral posterior neurons (LPN) (*14*). Recent single-cell sequencing has shown that these anatomical classifications do not capture the diversity of these neurons, single-cell RNA sequencing further divided *Drosophila* clock neurons; there are now 17 different groups without even considering all the DN3s (*15*, *16*).

Over the last few decades, the functions of many *Drosophila* clock neurons have been extensively studied. For example, the adult s-LNvs control morning activity and are essential for free-running rhythms in constant darkness (*17*). In contrast, LNds and non–pigment-dispersing factor (PDF)-expressing s-LNvs modulate both morning and evening activity (*18*, *19*). Both sleep and activity-promoting neurons were both identified within the DN1p population (*20*–*24*). In contrast, the gene expression and functions of almost all of the ~80 DN3s, about 70% of clock neurons per hemisphere, remain unknown. The singular exception is a recent study suggesting that a small subset of DN3 neurons receive signals from DN1 circadian neurons and then project to “claw” neurons to promote sleep (*24*, *25*).

In this study, we developed a split GAL4 driver line that is expressed in almost all of the DN3 neurons. We used this driver to drive GFP expression in DN3 neurons, allowing them to be isolated for droplet-based single-cell RNA sequencing method. DN3 neurons were collected and their transcriptomes were sequenced at six time points throughout the day. The DN3 neurons were separated into 12 different clusters by an unsupervised clustering algorithm. Some DN3 clusters had notable and unprecedented core clock gene expression patterns; these clusters have poor clock gene transcript cycling and a minimal number of cycling transcripts. Moreover, we show that TrissinR expression in several previously uncharacterized DN3 neurons promotes the sleep behavior of flies.

## RESULTS

### A complete high-resolution circadian network transcriptomic cell type atlas

To profile previously uncharacterized DN3 clock neurons, we screened new split GAL4 driver lines. In brief, carefully chosen activation domains (ADs) and DNA binding domains (DBDs), driven by two different regulatory regions, were combined so that a functional GAL4 protein was reconstituted only in the overlapping expression regions. The split GAL4 system offers greater specificity compared to the regular GAL4 lines (*26*). We found that the combination of R11B03-AD, from *clockwork orange* (*cwo*) and VT002670-DBD, from vrille (*vri*) identifies at least 50 to 60 clock neurons in the DN3 region of the fly brain, i.e., most of the 85 DN3 clock neurons reported in Nils *et al.* (*13*). It also labels the two DN1a, seven DN1p neurons and the s-LNvs with very minimal ectopic expression (fig. S1A). To our knowledge, this is the most comprehensive DN3 driver developed to date, which we will refer to as split-DN3-broad for simplicity.

**Fig. 1.**
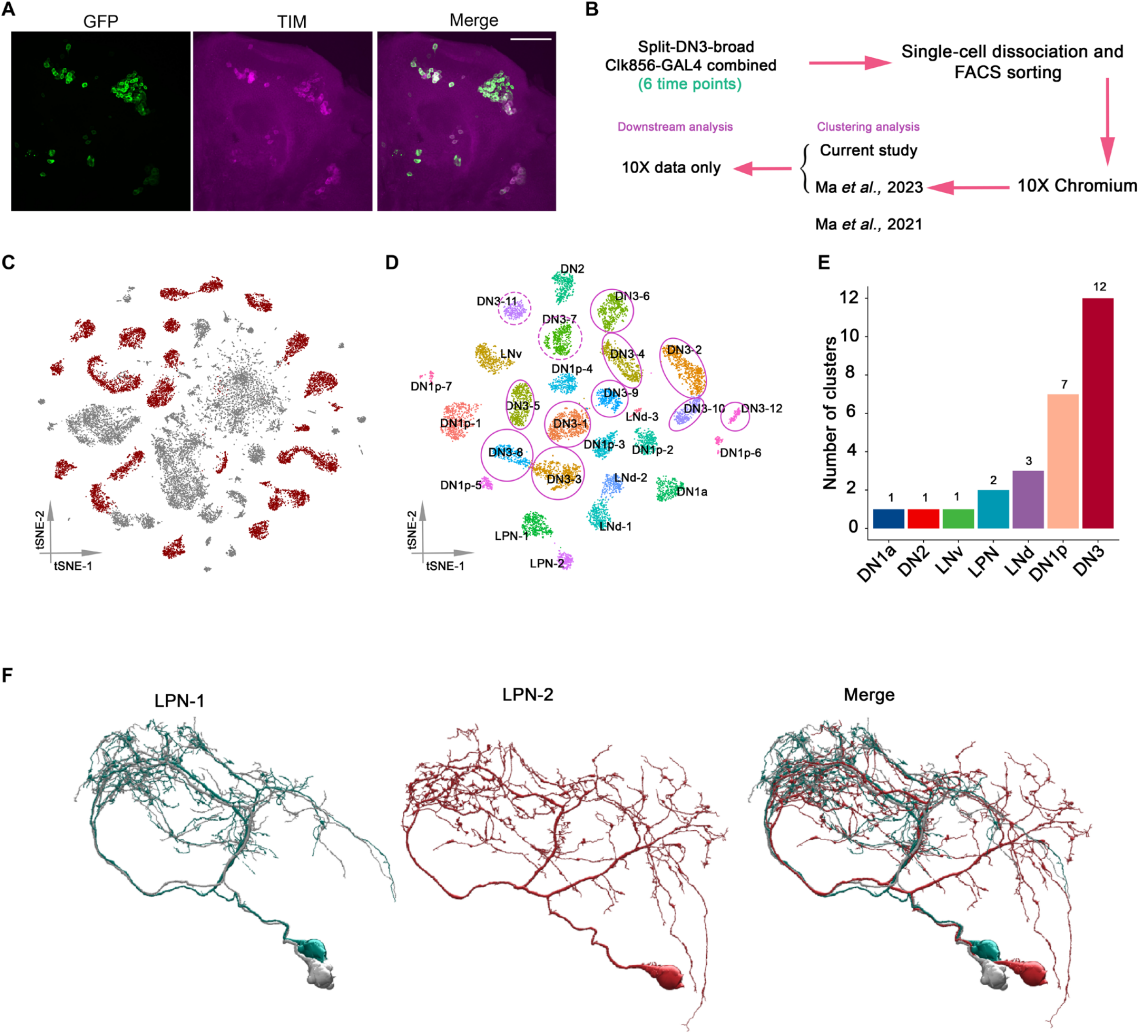
Transcriptomic taxonomy of all *Drosophila* clock neurons. (**A**) Confocal stack images of immunostained brains from Clk856-GAL4, Split-DN3-broad > UAS-Stinger-GFP flies at ZT18. Anti-GFP (left), anti-TIM (middle), and a merge of these two images (right). Scale bar, 50 μm. (**B**) Schematic workflow of single-cell RNA sequencing around the clock. Split-DN3-broad was crossed with a stable line of Clk856-GAL4 > UAS-EGFP, the flies were entrained for at least 3 days in LD condition before dissection. The single-cell RNA sequencing libraries were generated by 10X Chromium. High-confidence single cells were coclustered with the clock neurons identified previously. (**C**) t-distributed stochastic neighbor embedding (t-SNE) plot showing the 26210 cells grouped into 69 clusters. High-confidence clusters are shown in purple. (**D**) t-SNE plot of 10388 *Drosophila* clock in 27 high-confidence clock clusters. The clusters are colored by their cell types. (**E**) The number of transcriptomic cell types from different anatomic locations. The arrows indicate the LPN and DN3 clock neurons, respectively. (**F**) Electron microscopy (EM) reconstructions of three LPN clock neurons from the hemibrain dataset.

We next combined the split-DN3-broad with Clk856-GAL4, which has been used in our previous single-cell RNA sequencing studies (*15*, *16*). The combined driver line was then used to drive a nuclear localized enhanced green fluorescent protein (EGFP) expression. There were very few central brain ectopic cells as most of the GFP-expressing neurons were also Timeless (TIM) positive (Fig. 1A). This strategy allowed us to investigate universal clock neuron gene expression including the DN3s. Moreover, it enabled us to compare DN3 gene expression to previously characterized clock neurons side by side with very limited batch effect issues.

To profile these clock neurons, flies were entrained in a 12:12 light:dark (LD) cycle conditions for at least 3 days and then collected every 4 hours throughout the day. Dissected brains were dissociated in papain and triturated to achieve a single-cell suspension before being subject to fluorescence-activated cell sorting (FACS). The single-cell RNA sequencing libraries were generated by a commercially available high throughput droplet-based method (10X Chromium). At each time point, between 2631 and 6913 single cells were assayed (fig. S1B). To focus on the high-confidence single cells, we first applied a cell-wise filtering with the following cycling cutoffs: the number of detected transcripts was between 1000 and 25000, the number of detected genes between 300 and 3500, an entropy of gene expression greater than 5, and a percentage of mitochondrial RNA of less than 10%. After this stringent filtering, we had 9459 high-confidence single cells for our clustering analysis. We next combined these high-confidence single cells with previously characterized clock neurons, which were similarly subjected to LD entrainment (Fig. 1B and fig. S1C). The combined dataset not only gave us more statistical power, but the previously identified clock neuron clusters also served as a benchmark for the previously unidentified clustering.

The average number of detected genes and transcripts in our combined dataset were 1799 and 7304, respectively (fig. S1D). An unsupervised clustering algorithm was used to integrate the single-cell data from the different time points (*27*), resulting in 69 distinct clusters (Fig. 1C). A subsequent cluster-wide filtering based on core clock gene expression resulted in a final total of 27 high-confidence clock neuron clusters. Single cells from our earlier CEL-seq2 data and these 10X data were coclustered (fig. S1, E and F).

To assign cell identities to the different clusters, we compared previously known single-cell data with cells from the current study. Many clusters show a high degree of correspondence with previously known single cells, suggesting that these clusters were highly reproducible in our current study (fig. S1G); the Trissin-expressing LNds were an exception (see Discussion). De novo clustering analysis without accessing previous single-cell data was similar (fig. S2). On the basis of this information, we assigned cell identities to all 27 clusters (Fig. 1D). There are 10 previously unidentified clusters: They only contain cells only from this current study and reflect DN3 neurons never previously characterized. Combined with the two DN3 clusters previously identified, this makes a total of 12 DN3 clusters and 15 non-DN3 clock neurons clusters (Fig. 1E).

To rule out possible complications from combining more limited CEL-Seq2 data and the 10X data containing the 10 previously unidentified DN3 clusters, we focused only on the 10X data for subsequent analyses. There are cells from six time points in all these clusters (fig. S3). There is also a unique combination of marker gene expression associated with each cluster (fig. S4A and data S1), and previously known cell type–specific marker genes, for example, *Pdf*, *ITP*, and *CCHa1*, are all expressed in a cell type–specific manner (fig. S4B). This further increases our confidence in the clustering assignments.

There are three LPN neurons in each hemisphere of the fly brain (*28*). Although they were previously in a single cluster (*15*, *16*), the current single-cell data separated them into two different clusters. The numbers of cells in these two clusters are 457 and 234, respectively, correspond to a 2-to-1 ratio (fig. S5). This molecular characterization is notably consistent with the electron microscopy (EM) dataset and the morphology described by Reinhard *et al.* (*29*) (Fig. 1F): The two sleep-promoting cells in the LPN_1 cluster have very similar projections; the wakefulness-promoting single LPN_2 neuron has a different pattern characterized by bifurcated branches (*24*, *29*–*31*). Differential gene expression analysis further revealed that transcripts encoding the cell surface molecule side-II are enriched in the LPN_1 cluster, whereas beat-VII and the dopamine receptor Dop2R transcripts are more highly expressed in the LPN_2 cluster (fig. S6).

### Mapping DN3 clock neuron gene expression subtypes

**Fig. 2.**
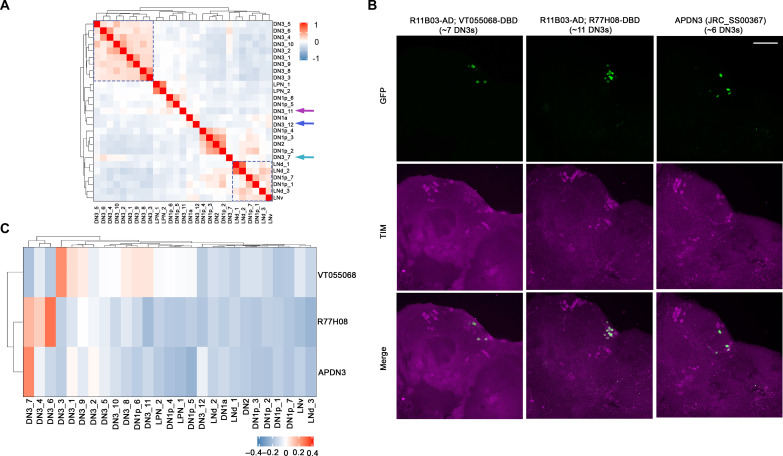
Mapping single-cell clusters to DN3 clock neurons. (**A**) Gene expression correlation of clock neuron clusters. Many of them show higher gene expression correlation except the three DN3 clusters pointed by colored arrows. (**B**) Expression pattern of sparse DN3 split GAL4 drivers. The driver lines were crossed with UAS-Stinger-GFP, flies were entrained in LD for 3 days before dissection at ZT18. Fly brains were costained with anti-GFP (green) and TIM (magenta). Scale bar, 50 μm. (**C**) Gene expression correlation between single-cell clusters and DN3 subgroups labeled by different sparse drivers. Flies were entrained in LD for 3 days and collected at ZT02.

To examine the differences in gene expression similarity between clock neuron clusters, we carried out gene expression correlation analysis. The LNd, LNv, and two DN1p neuron clusters show higher gene expression correlation (Fig. 2A, bottom right), in agreement with a previous result (*15*). Notably, most of the DN3 neuron clusters also have very similar gene expression (Fig. 2A, top left); the exceptions are DN3_7, DN3_11 and DN3_12 (Fig. 2A, purple arrows). As noted above, DN3_7 and DN3_11 were identified previously, indicating that they represent the DN3 neurons, which are also labeled by the Clk856-GAL4 driver.

To further map the DN3 single-cell clusters to brain neurons, we identified that two sparser DN3-relevant split GAL4s, R11B03-AD; VT055068-DBD and R11B03-AD; R77H08-DBD, label very specifically only 7 and 11 DN3 neurons, respectively (Fig. 2B). To assess the gene expression profiles of specific DN3 neurons, these previously unidentified DN3 driver lines and driver SS00367, which labels sleep-promoting APDN3 neurons (*25*), were used to drive EGFP expression. Cells were collected at ZT02, RNA sequencing libraries were generated with Smart-seq3 (*32*), and these bulk RNA sequencing data were compared to the single-cell data (Fig. 2C).

The analysis indicates that the APDN3s are most highly correlated with a single cluster, DN3_7. The DN3 neurons identified by R11B03-AD; R77H08 (split-DN3_4/6/7) correspond to three clusters: DN3_7 (APDN3s), DN_4, and DN3_6. R11B03-AD; VT055068-DBD (split-DN3_3) was most highly correlated with DN3_3 and more weekly with several other clusters (Fig. 2C).

### Unprecedented rhythmic gene expression in DN3s

**Fig. 3.**
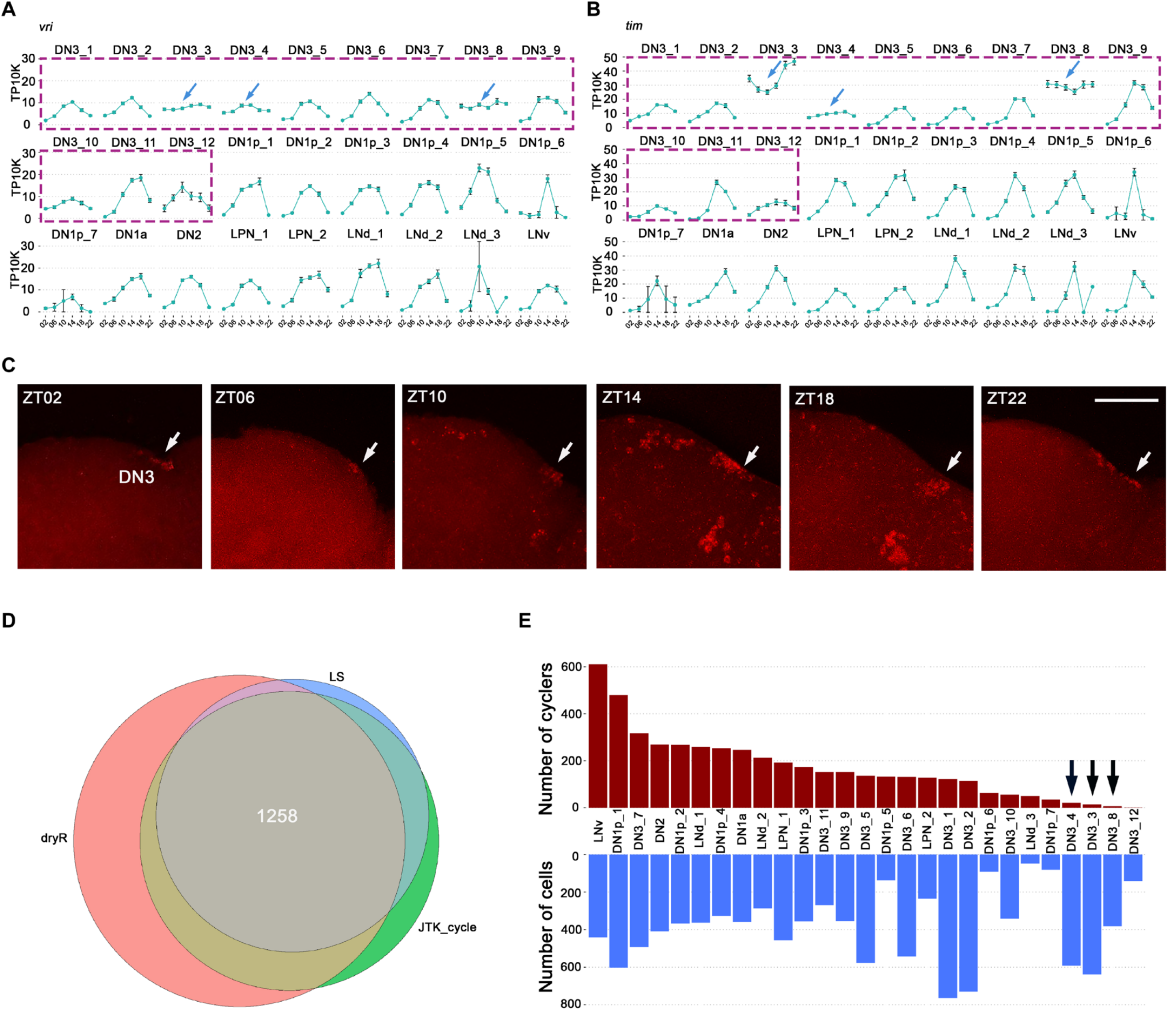
Unprecedented *tim* expression in DN3 neurons. (**A** and **B**) The mean *vri* (A) and *tim* (B) expression throughout the day in light: dark (LD) condition is graphed for each cluster. Error bars represent mean ± SEM. The arrows indicate the three DN3 clusters showing shifted or dampened *vri* or *tim* expression. (**C**) Fluorescent in situ hybridization (FISH) for *tim* mRNA transcripts in DN3 neurons throughout the day in LD condition. (**D**) Venn diagram showing the identified cycling transcripts by JTK_Cycle, LS, and dryR. There are 1258 high-confidence cycling transcripts in total. (**E**) The number of cells (bottom) and cycling transcripts in each cluster (top). The black arrows indicate the three DN3 clusters showing very limited rhythmic gene expression.

We suspected that the DN3 clock neuron clusters wound have rhythmic gene expression patterns similar to the other clock neurons clusters already characterized (*15*). The clock gene *vri* shows a canonical temporal pattern in most of the DN3 clusters with peak expression between ZT14 and ZT18 (Fig. 3A). However, three DN3 clusters have unprecedented *vri* patterns: The cycling amplitude of DN3_3, DN3_4, and DN3_8 is much reduced with substantial expression at trough times, early morning, and light night; moreover, the very modest peak of *vri* expression in DN3_3 is delayed relative to the peak time of other clusters (Fig. 3A, blue arrows). The *pdp1* cycling patterns in these three exceptional clusters are similar (fig. S7, blue arrows). The *tim* cycling patterns in two of these clusters, DN3_3 and DN3_8, are even more unusual than their *vri* and *pdp1* patterns; these two *tim* patterns show exceptionally high trough expression levels. Moreover, the delayed *tim* expression peak in DN3_3 is even more apparent than its delayed *vri* expression peak (Fig. 3B), which indicates that delayed clock gene expression in DN3_3 is probably general.

To further verify the unusual clock gene expression patterns in DN3 neurons, we carried out single molecular fluorescence in situ hybridization (FISH) in cleared whole-mount adult *Drosophila* brains (*33*). *tim* mRNA is clearly detectable only in the DN3s at traditional trough times of day (Fig. 3C), further underscoring the have unusual clock gene expression features of some DN3 neurons.

To address rhythmic gene expression of DN3s more generally, we used three standard algorithms to define oscillating gene expression among the clock neuron clusters: JTK_Cycle, Lomb-Scargle (LS), and dryR (*34*–*36*). The following cycling cutoffs were used to focus on robust cycling transcripts: a JTK_cycle *P* value of less than 0.05, a LS *P* value of less than 0.05, a dryR *P* value of less than 0.0, a cycling amplitude (maximum expression divided by minimum expression) of at least twofold, and a maximal expression of at least 0.5 TP10K. In total, 1258 cycling transcripts were identified within the clock neuron clusters by these three different methods (Fig. 3D).

The number of cycling transcripts varies greatly between clusters, due in large part to the variable number of recovered cells per cluster. Notably, however, DN3_3, DN3_4, and DN3_8 have many fewer cycling transcripts than DN3_7 and DN3_11 despite the fact that the first three clusters have more cells than DN3_7 and DN3_11 (Fig. 3E). In light of the aberrant clock gene cycling profiles expression in DN3_3, DN3_4, and DN3_8, we speculate that the molecular circadian program in these three clusters is less robust than in other circadian neurons clusters (see Discussion).

Activity-regulated genes (ARGs) are typically rapidly induced by neuronal firing presumably without the need for de novo protein synthesis and with important roles in neuronal function. We next investigated ARG expression in our clock neuron subtypes to explore whether they could reflect neuronal activity patterns across the day. Consistent with previous genome-wide analysis in the fly brain and sorted neurons (*37*), ARG expression is remarkably cell type specific at the single-cell level. Among the known ARGs, *sr*, *Hr38*, *CG14186*, and *CG17778* are rhythmically expressed in several clock neuron clusters, but their peak expression phases vary (fig. S8). These expression patterns suggest that ARGs respond in clock neuron–specific ways and perhaps even to different stimuli.

**Fig. 4.**
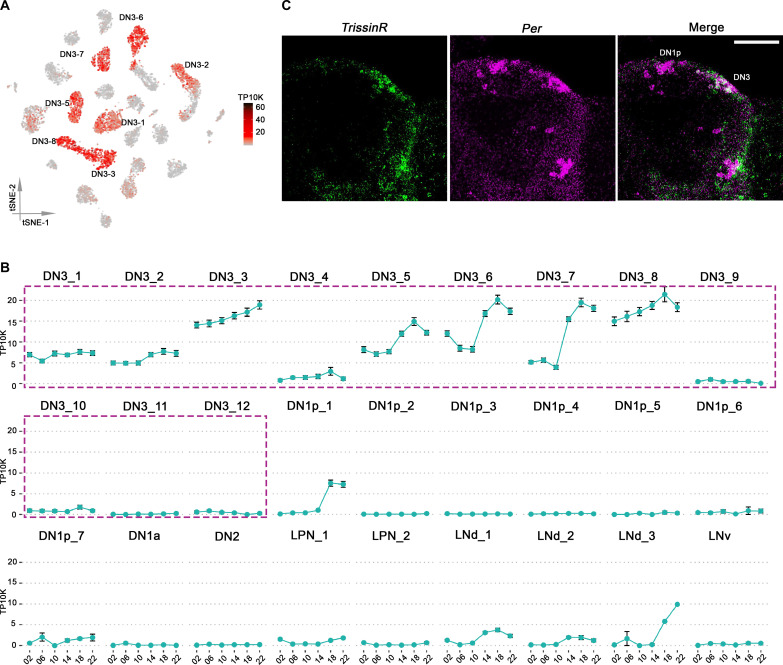
*TrissinR* expression in Drosophila clock neurons. (**A**) t-SNE plots showing *TrissinR* expression. Red indicates higher expression (color bar, TP10K). (**B**) The mean *TrissinR* expression throughout the day in LD condition is graphed for each cluster. Error bars represent mean ± SEM. *TrissinR* expression is specific to the DN3 neurons. (**C**) RNA-scope for *TrissinR* mRNA transcripts in DN3 neurons at ZT18.

Neurotransmitters and neuromodulators interact with G protein–coupled receptors (GPCRs) to excite, inhibit, or modulate the GPCR-expressing neurons. Our recent studies have shown that GPCRs are highly enriched in the fly brain clock network, and GPCR transcripts alone can define the adult brain dopaminergic and circadian neurons (*16*, *38*). Notable in this context, *CapaR* encodes the GPCR for the neuropeptide Capa, and this transcript is substantially and rhythmically expressed in only a single DN3 cluster, DN3_11 (fig. S9). A second GPCR transcript, *TrissinR*, is highly expressed in seven different DN3 clusters (Fig. 4A) and oscillates robustly in clusters DN3_5, DN3_6, and DN3_7 (Fig. 4B). Its ligand, the neuropeptide Trissin, is expressed in only two LNd clock neurons and a few neurons outside of the clock network (*15*, *39*).

To further verify *TrissinR* expression in DN3s, we conducted in situ hybridization with *TrissinR* probes at ZT18, when TrissinR shows peak expression levels (Fig. 4B). *TrissinR* is indeed enriched in the DN3 region of the fly brain, which is further defined by *per* expression (Fig. 4C).

### TrissinR expression in DN3 subtypes promoting sleep

**Fig. 5.**
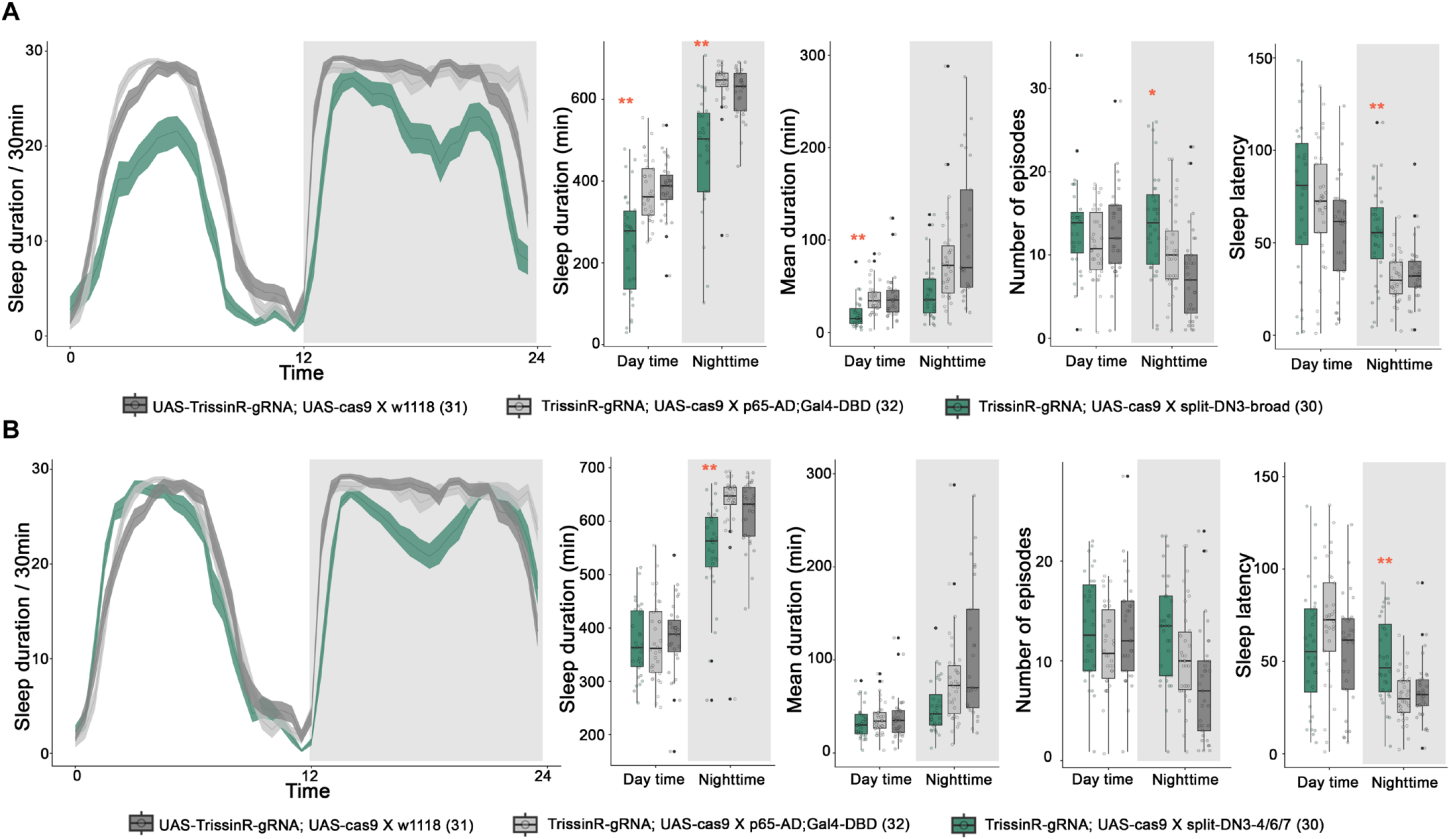
TrissinR expression in DN3 neurons promotes sleep. (**A** and **B**) Sleep plots of split-DN3-broad (A), split-DN3-4/6/7 (B) driven UAS-TrissinR-gRNA; UAS-Cas9 (blue), and controls (light and dark gray). The solid lines represent the averaged sleep amount, and the shading represents SEM for each time point. Quantified sleep durations, mean durations, number of sleep episodes, and sleep latency are shown on the right panels. An asterisk (*) represents *P* < 0.05, and two asterisks (**) indicate *P* < 0.01. Statistical analysis was performed using a one-way ANOVA with post hoc Tukey’s post hoc test.

To address a possible biological function of TrissinR expression in DN3s, we exploited our recent *Drosophila* GPCR gRNA library and recently characterized split GAL4 driver lines to knock out *TrissinR* only in DN3s (*38*). The DN3 driver split-DN3-broad resulted in a robust reduced sleep phenotype in the daytime and the nighttime (Fig. 5A). Knocking down TrissinR expression with the same driver and an RNAi line showed only reduced sleep during the nighttime, perhaps because the RNAi approach is less complete than the guide approach (fig. S6). To further investigate DN3 neuron subsets, we knocked out *TrissinR* only in the approximately 11 DN3 neurons expressed by the split-DN3_4/6/7 driver. These flies only showed reduced nighttime sleep with sleep latency also substantially increased (Fig. 5B). Given the much more modest effect of knocking out *TrissinR* with the APDN3 and split-DN3-3 drivers (fig. S11), as well as the expression patterns of the split-DN3_4/6/7 driver (Fig. 2C), much of the *TrissinR* effect may come from cluster 6.

## DISCUSSION

This study provides an invaluable resource, namely, the most comprehensive and high-resolution single-cell transcriptomic analysis of the 240 *Drosophila* clock neurons with a particular focus on the previously uncharacterized DN3s. This medium size number of clock network neurons makes it an ideal system to study how gene expression patterns within different transcriptomic cell types contribute to distinct projection patterns and ultimately a variety of circadian behaviors. Gene expression very likely underlies the heterogeneous morphologies of different clock neurons. Moreover, different DN3 subtypes regulate the sleep behavior of flies in a time-dependent manner.

It may appear surprising that the about 120 *Drosophila* clock neurons (in each hemisphere) are separated into so many, at least 27 distinct transcriptomic cell types. However, we previously showed that most clock neurons and even adult fly brain dopaminergic neurons are more heterogeneous at the RNA level than anatomically defined (*15*, *16*), and this current study extends this conclusion to the enigmatic DN3 clock neurons. There are at least 12 DN3 transcriptomic cell types of which two were previously identified (*15*). This is in contrast to the seven anatomical types identified by Nils *et al.* (*13*). Nonetheless, the large number of DN3 neurons suggests that they may be less heterogeneous at the RNA level than the more traditionally defined clock neurons. Perhaps many DN3 neurons emerge together at a late stage of development and share a more closely related linage than other clock neurons. It is of future interest to compare in more detail gene expression features of different DN3 cell types and how they contribute to function as we have done here for *TrissinR*.

The large number of gene expression cell types still somewhat underrepresents the diversity of gene expression within clock neurons. Although most of the previous single-cell transcriptomic cell types were reproduced in the current study, we failed to identify the Trissin-expressing LNd neurons, which seem particularly sensitive to these single-cell RNA sequencing methods. These cells were reliably detected only with modified CEL-Seq2 method, but not the 10X Chromium, perhaps due to the difference in the underlying sequencing library preparation biochemistry between these two methods. Nonetheless, the remarkable gene expression diversity described here contrasts with the single-cell/nucleus RNA sequencing efforts from mice SCN (*6*–*8*).

In addition to the gene expression classification of clock neurons, our study also allows us to compare the transcriptomic cell types with neuron morphologies and projection patterns. The six LNd clock neurons were separated into four different morphological groups, which are in concordance with the EM dataset (*15*, *40*). The three LPN neurons are now separated into two distinct clusters, which is likely due to the excellent cell coverage provided by the current study and reflecting the high fidelity of our single-cell gene expression analysis. It is of great interest how these two adjacent LPN groups interact with their partners to regulate sleep and wakefulness, respectively. Because the single-cell data in the current study were only assayed from adult flies, the genes involved in neuronal differentiation and axon guidance during development may not be represented in the data. This makes gene expression in developing clock neurons of future interest.

Most of the lateral clock neurons and some of the dorsal clock neurons have been extensively studied, but the gene expression basis and functions of DN3 clock neurons are largely unknown. One main reason for this deficiency is the lack of good driver lines to label these neurons. The DN3 neurons appear to be heterogeneous based on previous immunohistochemistry studies (*29*, *41*, *42*). The joint effort of split GAL4 screening and single-cell RNA sequencing in the current study resolved the gene expression basis of DN3 clock neuron identity for the first time in decades. The methodology in the current study is readily applicable to other neurons in *Drosophila* adult brain.

Rhythmic gene expression in the brain and peripheral tissues dictates many different behaviors and at least 80% of protein-coding genes are reported to be oscillating in a daily manner in a tissue-specific fashion (*43*, *44*). In this context, some DN3 clusters had unusual cycling features, i.e., their core clock gene expression was shifted or showed a dampened amplitude. More general rhythmic gene expression analysis also indicated that there were very few cycling transcripts in these clusters, especially in DN3_3, DN3_4, and DN3_8. We noted that the two DN3 clusters labeled by Clk856-GAL4 showed more robust core clock gene cycling and cycling transcript expression in general. How core clock gene expression is differentially regulated in these neurons needs further investigation.

GPCRs play a pivotal role in neuron communication. Our previous studies showed that GPCRs are enriched in *Drosophila* clock neurons, and GPCRs alone could define the cell types within the clock network (*16*, *38*). Indeed, a large number of GPCRs are rhythmically expressed in a cell type–specific manner, which further underscores the importance of the dynamic interactions between the circadian network and their upstream or presynaptic partner neurons. In addition, our analysis of cell type–specific GPCR expression provides functional insight into DN3 function. We show that TrissinR expression in different DN3 subtypes promotes fly sleep. These findings complement our previous study showing that a small subset of DN3 neurons receives synaptic input from DN1ps and project to claw neurons to promote fly sleep (*25*).

It is notable that Trissin expression in the brain is highly specific. In addition to the two Trissin-expressing circadian LNd neurons identified on the basis of a fly chemoconnectome line (*15*, *39*, *45*), Trissin is also enriched in another uncharacterized neuron in the fly midbrain based on a chemoconnectome Trissin-Gal4 line (fig. S12). A recent study suggested that Trissin-expressing LNds promotes the transition from arousal to sleep at dusk (*46*).

In summary, the DN3 clock neurons in *Drosophila* adult brain are notably heterogeneous in gene expression and function. Moreover, the developed driver lines and many cell type–specific marker genes identified in this study will help drive further understanding of DN3 clock neuron function.

## MATERIALS AND METHODS

### Fly strains and rearing

Flies were raised on standard cornmeal medium with yeast under LD (12-hour light/12-hour dark) conditions at room temperature. The fly lines used in this study are listed in the key resource table.

### Fluorescence-activated cell sorting

To label the clock neurons, we first generated a stable line of Clk856-GAL4; UAS-EGFP and then crossed it with R11B03-AD; VT2670-DBD. Equal numbers of males and females were first entrained for at least 3 days under LD conditions at 25°C before dissection at different time points of the day. Fly brains were dissected in cold dissection saline [9.9 mM Hepes KOH (pH 7.4), 137 mM NaCl, 5.4 mM KCl, 0.17 mM NaH_2_PO_4_, 0.22 mM KH_2_PO_4_, 3.3 mM glucose, and 43.8 mM sucrose] with neuronal activity inhibitors (20 μM 6,7-dinitr oquinoxaline-2,3dione, 0.1 μM tetrodotoxin, and 50 μM d,l-2-amino-5-phosphonovaleric acid). The brains were digested with papain (50 U/ml; ~2 μl per brain; Worthington Biochemical, #LK003176) at room temperature for 25 to 30 min. Brains were then washed twice with ice-cold active Schneider’s Medium after the digestion. Flame-rounded 1000-μl pipette tips with different-sized openings were used to triturate the brains. The resulting cell suspension was passed through a 100-μm sieve. Hoechst dye (one drop per 0.5 ml of sample; Invitrogen, #R37605) was added into the sample tube to stain the nucleus (15 min at room temperature). A BD Melody FACS machine in single-cell sorting mode was used for cell collection. GFP- and Hoechst-positive single cells were collected in a 1.5-ml Eppendorf tube containing 0.3 ml of collection buffer [phosphate-buffered saline (PBS) + 0.04% bovine serum albumin] and used for downstream single-cell RNA sequencing library preparation. To minimize the possible stress to the cells, the collection devices were kept at 5°C constantly during the sorting process.

### Library preparation and raw data processing

The collected cells from the FACS machine were spun down on a benchtop centrifuge by 700*g* for 10 min at 4°C before loaded to the GEM chip from the Chromium Single Cell 3′ Kit (version 3) of 10X Genomics. The libraries were prepared according to the standard user guide (CG000315 Rev. B) from 10X without any modifications. 10X libraries were sequenced by Illumina NextSeq 500 with the High Output Kit version 2.5 (75 cycles). The Cell Ranger package from 10X was used to map the sequencing data to the *Drosophila* genome (dm6), only the alignments to annotated exons were used for unique molecular identifier (UMI) quantitation.

### Dimensionality reduction and clustering analysis

To focus on the high-confidence single cells, we first filter out low-quality cells based on the following criteria: (i) fewer than 300 or more than 3500 detected genes; (ii) fewer than 1000 or more than 15000 total UMI; (iii) gene expression entropy smaller than 5.0, where entropy was defined as −nUMI x ln(nUMI) for genes with nUMI >0, where nUMI was a number of UMI in a cell; and (iv) the percentage of mitochondria RNA greater than 10%. The possible doublets were detected by Scrublet with default setting, and excluded from the downstream analysis.

We next combined the high-confidence clock neurons from previous studies and the single cells from current study to identify the facile clock neuron clusters (*15*, *16*). The integration and clustering method has been described previously (*9*). We first separate the raw dataset by methods and time points, then carry out data transformation, normalization and variance by SCTransform function from the Seurat package (*27*). The batch effect was removed by regressing out numbers of genes, UMIs, detected genes per cell, sequencing batches, and percentage of mitochondrial transcripts. We computed 3000 variable genes at each time point and method and only used the shared variable genes in all conditions for the clustering analysis, which resulted in 69 clusters. We next applied a cluster-wise filtering; only the clusters with high core clock genes were retained.

### Bulk RNA sequencing

Flies were entrained for at least 3 days before being collected and dissected at ZT02. GFP-positive neurons were sorted and collected as aforementioned. PolyA mRNA was isolated with the Dynabeads mRNA direct kit (Thermo Fisher Scientific 61011). Subsequently, cDNA and sequencing libraries were prepared using Smart-seq3 (*32*). Final libraries were quantified on a High Sensitivity D1000 ScreenTape on the Agilent TapeStation.

### Cycling transcripts analysis

We used three different computational methods, including JTK_Cycle, LS, and dryR, to identify the facile cycling transcripts in single-cell clusters (*34*–*36*). To be considered cycling, the following cutoffs were used: (i) a JTK_cycle *P* value of less than 0.05, (ii) an LS *P* value of less than 0.05, (iii) a dryR *P* value of less than 0.01, (iv) a cycling amplitude (maximum expression divided by minimum expression) of at least twofold, and (v) a maximal expression of at least 0.5 TP10K.

### Immunostaining

Three to 5-day-old flies were entrained in LD conditions for 3 days before being fixed with 4% (v/v) paraformaldehyde with 0.5% Triton X-100 for 2 hours and 40 min at room temperature. Brains were dissected and then washed twice (10 min) in 0.5% PBST buffer before being blocked overnight in 10% normal goat serum (Jackson ImmunoResearch Lab) at 4°C. The brains were then incubated in, a rat anti-TIM at 1:200 dilution, a mouse or chicken anti-GFP antibody at a 1:1000, a rat anti–red fluorescent protein antibody at 1:200 for overnight, then the brains, then were washed twice (10 min) in 0.5% PBST buffer. The corresponding secondary antibodies were added and incubated overnight. Brains were mounted in Vectashield (Thermal Fisher Scientific) and imaged on a Leica SP8 confocal microscope.

### Fluorescent in situ hybridization

FISH for *timeless* expression was performed as described previously onto wild-type flies (w1118) at different times of day with the following exceptions: custom oligo probes were ordered against the entire *timeless* mRNA sequence and conjugated with Quasar 670 dye (Stellaris Probes, Biosearch Technologies). The probes were diluted to a final concentration of 0.75 mM for the hybridization reaction. The brains were washed before mounting them onto slides with Vectashield Mounting Medium (Vector Laboratories). The slides were immediately viewed on a Zeiss 880 series confocal microscope.

### Drosophila behavior assay

We used the *Drosophila* Activity Monitoring System (Trikinetics) to record the number of beam crosses caused by the fly in 1-min intervals. Five- to 6-day-old male flies were used for the behavior analysis, in which flies were individually placed in glass tubes containing 2% agar and 4% sucrose. The flies were entrained under 12:12 LD conditions for at least 3 days. Each experiment was performed at least twice and got similar results. The sleep analysis was performed with MATLAB. Statistical analysis was performed using a one-way ANOVA (analysis of variance) with post hoc Tukey’s post hoc test (www.statskingdom.com), and a *P* value of less than 0.05 compared to all control groups was considered significant.
