# Supplementary material for: Transcriptomic DN3 clock neuron subtypes regulate Drosophila sleep
Source: Sci Adv. 2025 Jan 3;11(1):eadr4580. doi: 10.1126/sciadv.adr4580 (PMC11698076; doi:10.1126/sciadv.adr4580)
Supplement: Supplementary file 1 — Figs. S1 to S12 Table S1 Legend for data S1 [file sciadv.adr4580_sm.pdf]

Science Advances
AAAS

Supplementary Materials for

Transcriptomic DN3 clock neuron subtypes regulate Drosophila sleep

Dingbang Ma *et al.*

Corresponding author: Dingbang Ma, dbma@sioc.ac.cn; Michael Rosbash, rosbash@brandeis.edu

Sci. Adv. **11**, eadr4580 (2025)
DOI: 10.1126/sciadv.adr4580

The PDF file includes:

Figs. S1 to S12
Table S1
Legend for data S1

Other Supplementary Material for this manuscript includes the following:

Data S1

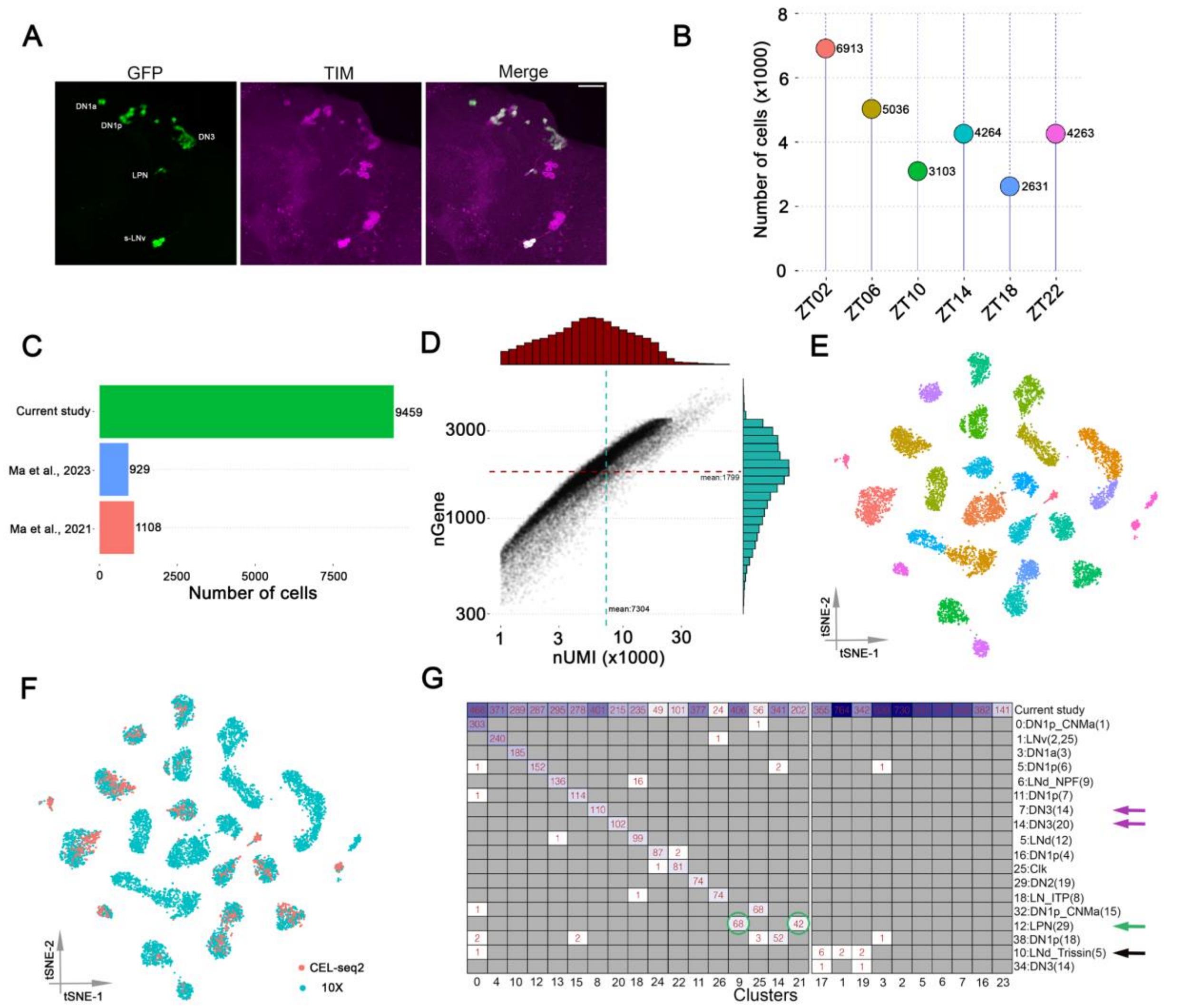

Figure S1. Identification of high confidence *Drosophila* clock neuron clusters. (A) Confocal stack images of immunostained brains from R11B03-AD; VT2670-DBD > UAS-Stinger-GFP flies at ZT18. Anti-GFP (left), anti-TIM (middle) and a merge of these two images (right). The scale bar represents 50 µm. (B) The number of single cells from 10X Chromium at each time point before initial filtering. (C) The number of high confidence single cells for the clustering analysis. (D) The mean detected genes and transcripts in the combined dataset. (E) t-SNE plot showing the 27 high-confidence clock clusters after the cluster wise filtering. The clusters are colored by their cell types. (F) CEL-Seq2 data and 10X data are co-clustered in different clusters. CEL-Seq2 data are shown in red and 10X data are shown in blue. Two previously identified DN3 clusters are

highlighted in circles. (G) Heatmap showing the high degree of correspondence of the single cells from previous and current studies. The arrows in magenta indicate two previously identified DN3 clusters, the arrow and circles in blue indicate the two LPN clusters, and the arrow in black indicate the Trissin-expression LNds.

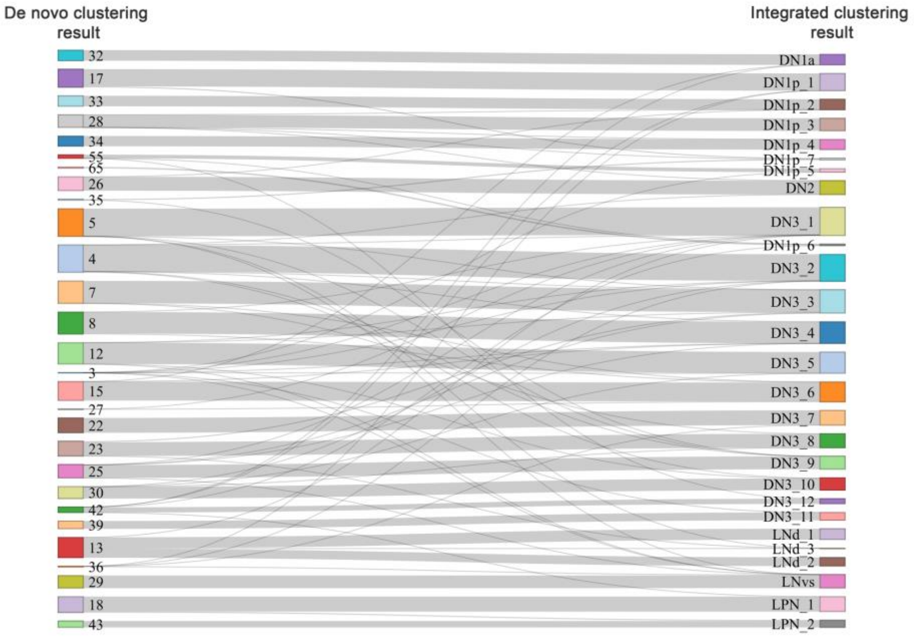

Figure S2. De novo clustering analysis of the single cell data from the current study. (A) Sanky plot showing the clustering result of the single cells from the current study only (left) compared to the integrated classification (right) analysis. Each node represents a single cell cluster.

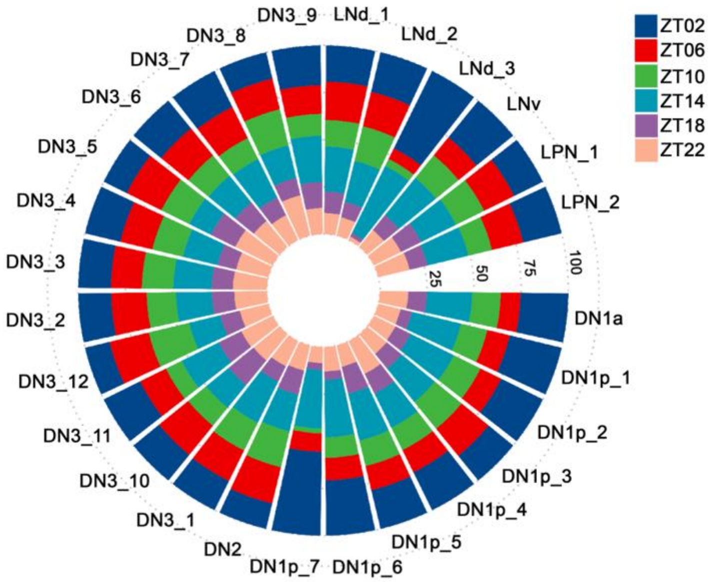

Figure S3. The percentage of cells from six time points in each single cell cluster. Circled bar plot showing that in high confidence clusters there are cells from 6 time points in Light: Dark conditions. Each time point is represented by different colors.

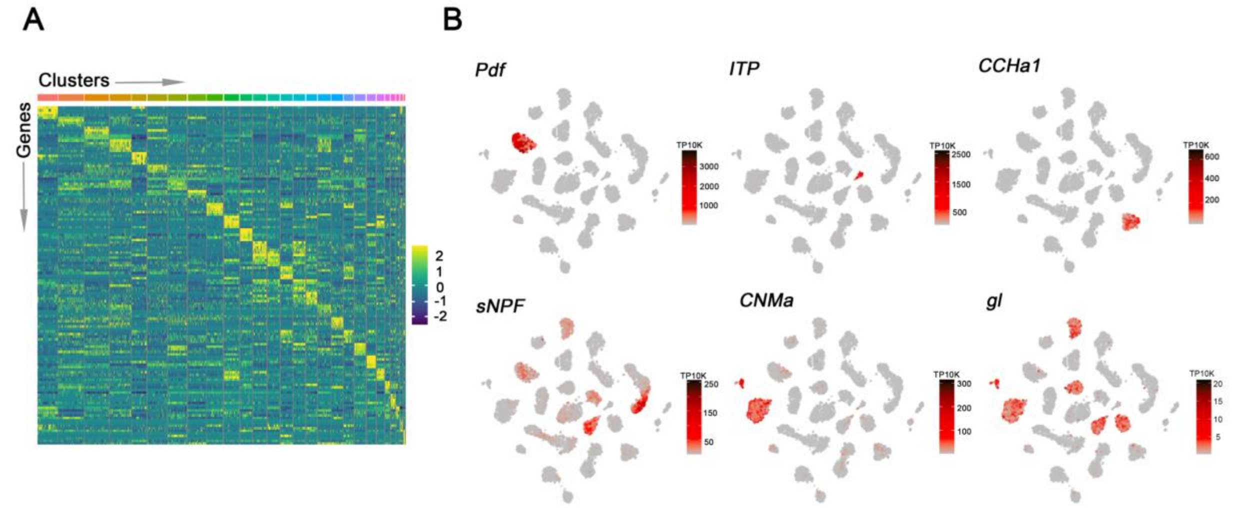

Figure S4. Marker gene expression in each cluster. (A) Heatmap showing the expression levels of the top 5 differentially expressed genes (rows) in cells (columns). Clusters are ordered by size and are represented by different colors on top of the heatmap. (B) t-SNE plots showing previously known marker gene expression in all clusters. Each cell is colored by the expression levels with gray indicating low expression and black indicating the highest expression.

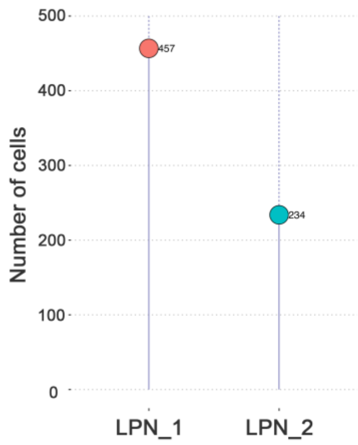

Figure S5. The number of cells in LPN_1 and LPN_2 clusters. The LPN_1 cluster contains 457 single cells, while the LPN_2 cluster has 234 single cells.

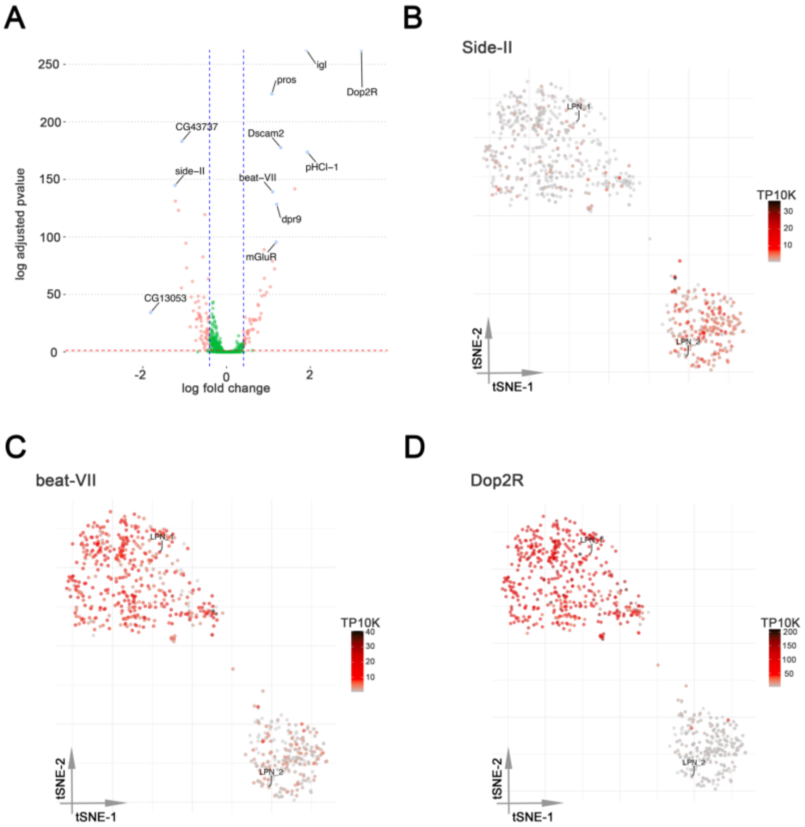

Figure S6. Differentially expressed genes in LPN_1 and LPN_2 clusters. (A) Volcano plot showing the overall differentially expressed genes (highlighted in red) between the two LPN clusters. (B-D) *Side-II* (B) is enriched in LPN_2, while *beat-VII* (C) and *Dop2R* (D) are highly expressed in LPN_1 cluster.

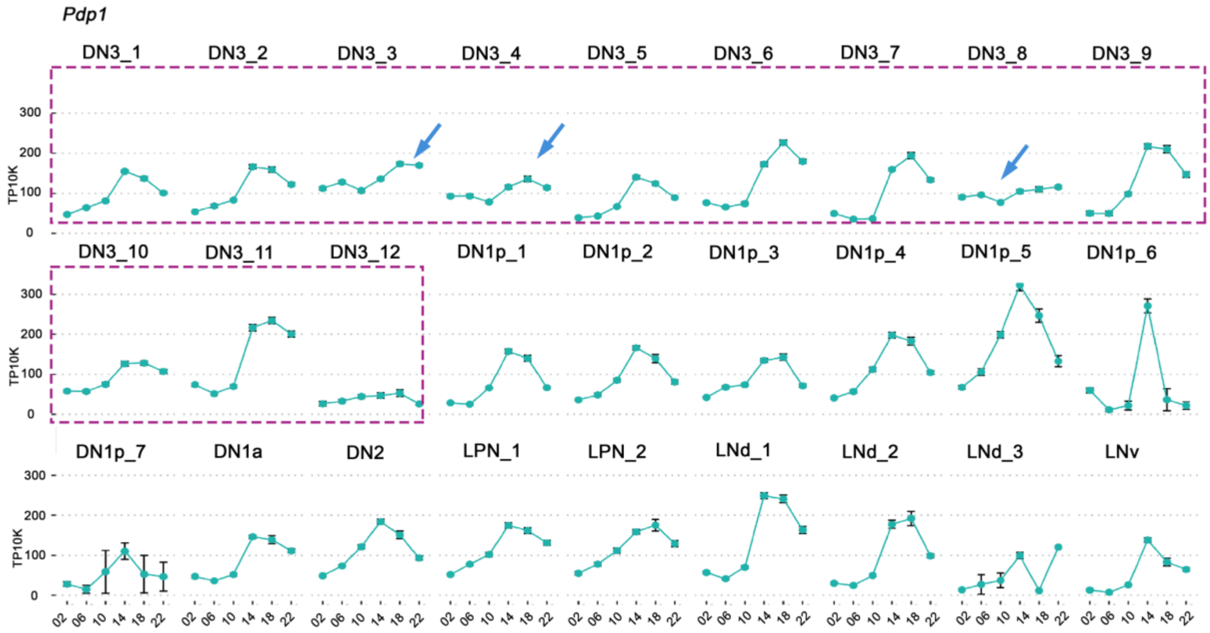

Figure S7. *Pdp1* expression in each cluster. The mean *Pdp1* expression throughout the day in LD condition is graphed for each cluster. Error bars represent mean ± SEM. The arrows indicate the three DN3 clusters showing shifted or dampened *Pdp1* expression.

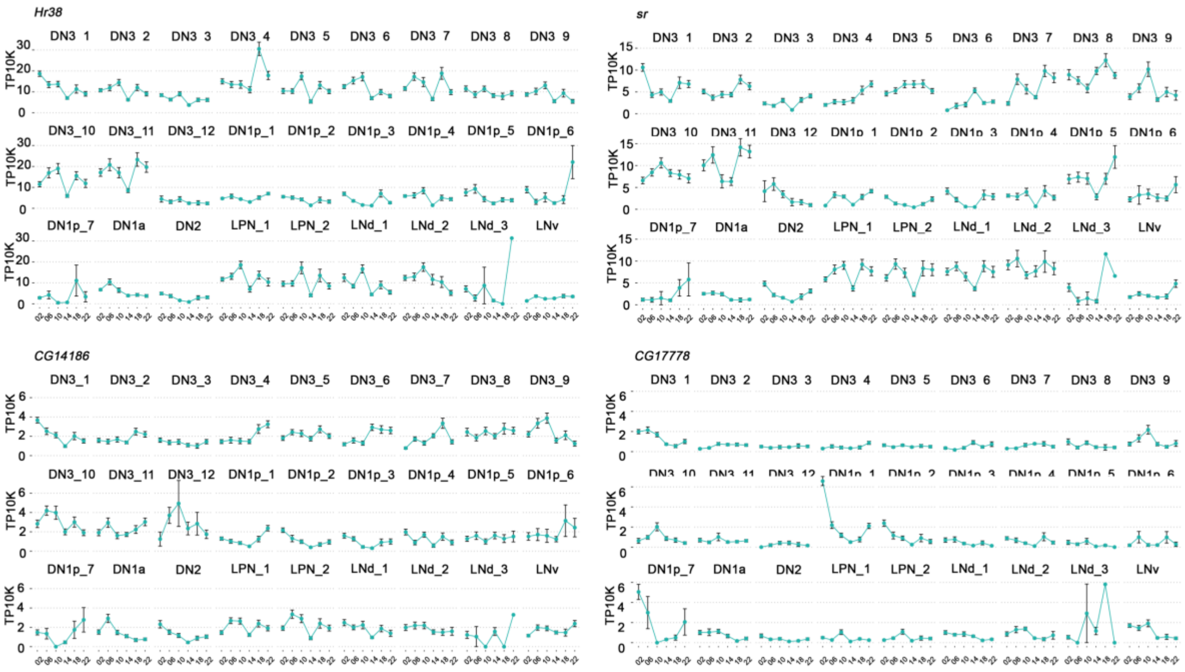

Figure S8. Representative expression of activity-regulated genes in each cluster. The mean *Hr38*, *sr*, *CG14186* and *CG17778* expression throughout the day in LD condition is shown for each cluster. Error bars represent mean ± SEM.

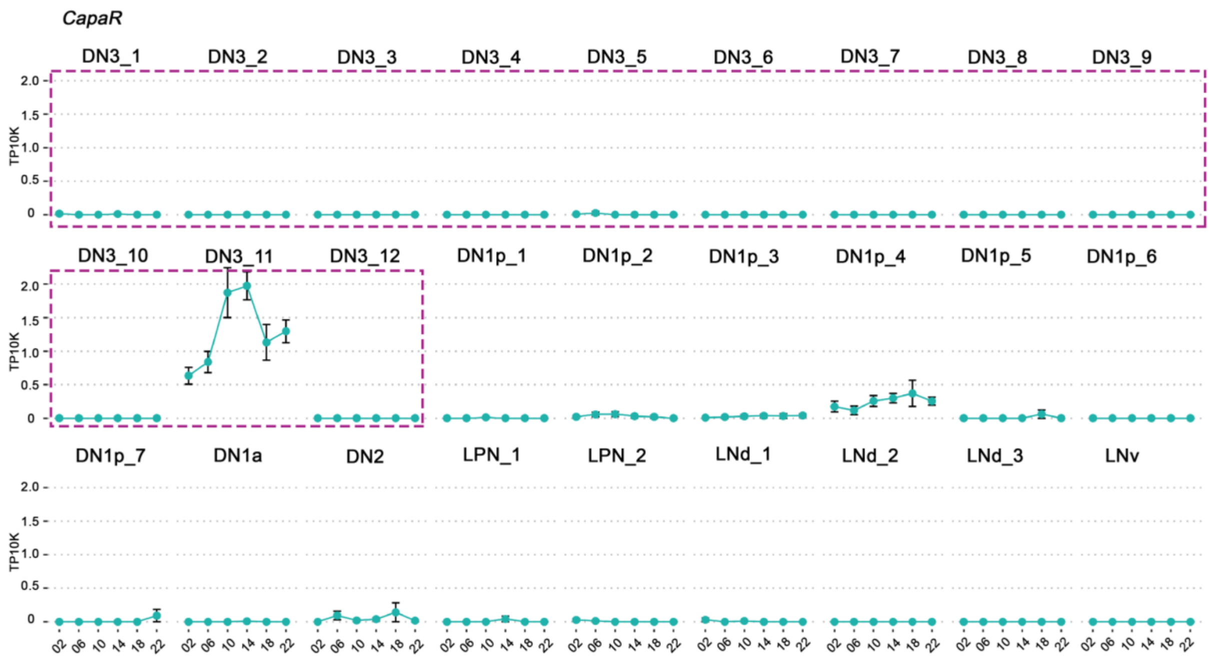

Figure S9. *CapaR* expression in each cluster. The mean *CapaR* expression throughout the day in LD condition is graphed for each cluster. Error bars represent mean ± SEM. The arrows indicate the three DN3 clusters showing shifted or dampened *CapaR* expression.

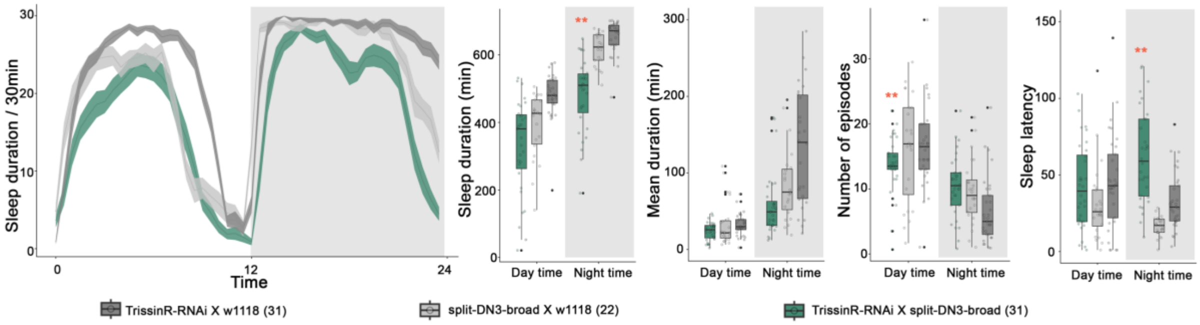

Figure S10. TrissinR expression in DN3 neurons promotes sleep. Sleep plots of split-DN3-broad driven UAS-TrissinR-RNAi (36825) and controls (gray). The solid lines represent the averaged sleep amount, and the shading represents SEM for each time point. Quantified sleep durations, mean durations, number of sleep episodes and sleep latency are shown on the right panels. An asterisk (*) represents P<0.05, and two asterisks (**) indicate P< 0.01. Statistical analysis was performed using a One-way ANOVA with post-hoc Tukey HSD test.

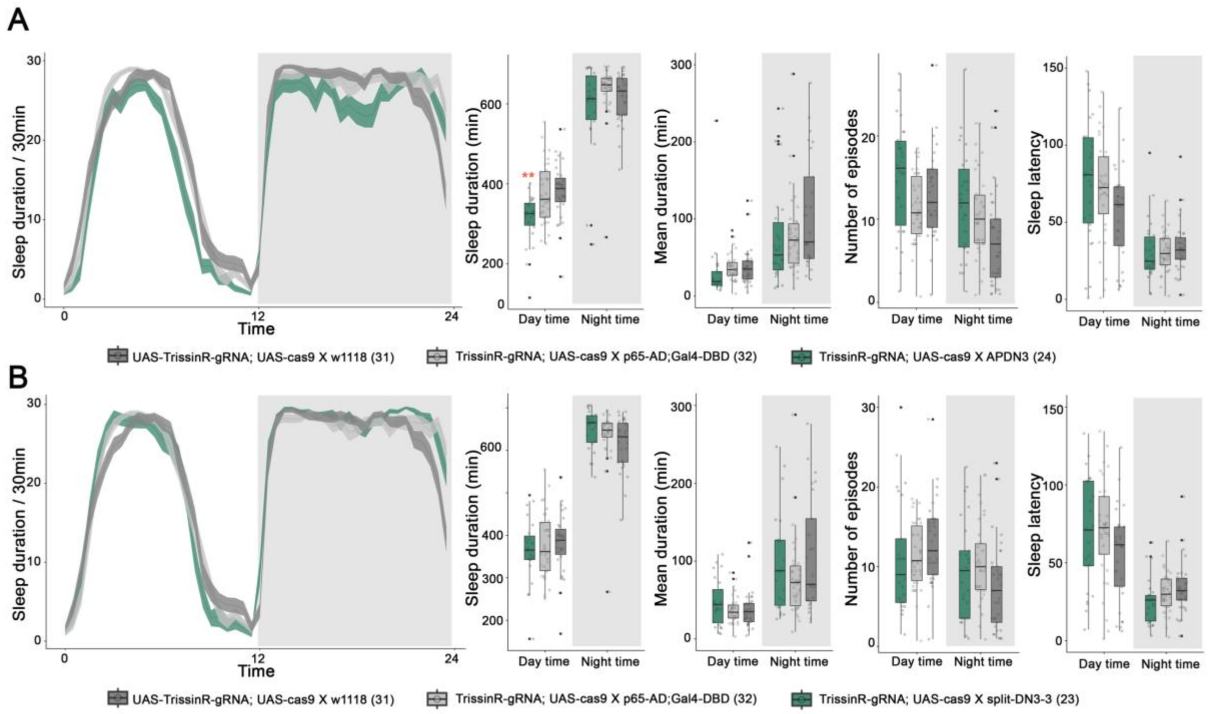

Figure S11. Knocking out TrissinR expression in APDN3 and cluster DN3_3 only resulted in modest effect. (A-B) Sleep plots of APDN3(A), split-DN3-3 (B) driven UAS-TrissinR-gRNA; UAS-Cas9 (blue) and controls (light and dark gray). The solid lines represent the averaged sleep amount, and the shading represents SEM for each time point. Quantified sleep durations, mean durations, number of sleep episodes and sleep latency are shown on the right panels. Two asterisks (**) indicate P< 0.01. Statistical analysis was performed using a One-way ANOVA with post-hoc Tukey HSD test.

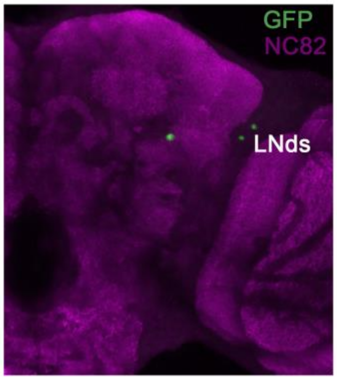

Figure S12. Trissin expression in *Drosophila* brain. Confocal stack images of immunostained brains from Trissin-Gal4 > UAS-H2A-GFP flies. In addition to the two LNd neurons, Trissin is also expressed in another neuron in the middle brain.

Table S1 - Key resources

Reagent types	Designation	Source or reference	Identifiers	Additional information
Genetic reagent (*D. melanogaster*)	UAS-Stinger	BDSC	RRID:BDSC_84277	
Genetic reagent (*D. melanogaster*)	Clk856-GAL4		Flybase: FBtp0069616	
Genetic reagent (*D. melanogaster*)	UAS-EGFP	BDSC	RRID: BDSC_5428	
Genetic reagent (*D. melanogaster*)	R11B03-p65.AD	BDSC	RRID: BDSC_70531	
Genetic reagent (*D. melanogaster*)	VT002670-DBD	BDSC	RRID: BDSC_74998	
Genetic reagent (*D. melanogaster*)	R77H08-DBD	BDSC	RRID: BDSC_69630	
Genetic reagent (*D. melanogaster*)	VT055068-DBD	BDSC	RRID: BDSC_75126	
Antibody	anti-GFP Chicken	Abcam	RRID: AB_300798	1:1000
Antibody	Goat anti-mouse polyclonal	ThermoFisher	RRID: AB_2536185	1:200
Antibody	Goat anti-Chicken polyclonal	ThermoFisher	AB_2534096	1:200
Software, algorithm	FIJI	https://fiji.sc/		
Software, algorithm	RStudio	https://rstudio.com	RRID: SCR_000432	

Data S1

A list of differentially expressed genes in each cluster.
